# Stabilization of canine ilial body fractures using an anatomically contoured locking iliac plate: A short retrospective case series

**DOI:** 10.1371/journal.pone.0352364

**Published:** 2026-11-10

**Authors:** Cassio Ricardo Auada Ferrigno, Vanessa Couto de Magalhaes Ferraz, Alessandra Chiaramonte, Greg Woo, Alexis Boone, Thomas Bevelock, Courtney Weaver, Leah Kasmark, Marcio Poletto Ferreira

**Affiliations:** 1 Small Animal Clinical Science, University of Tennessee College of Veterinary Medicine, Knoxville, Tennessee, United States of America; 2 Knox Veterinary Surgery, Knoxville, Tennessee, United States of America; 3 Federal University of Rio Grande do Sul, College of Veterinary, Porto Alegre, Brazil; Shuguang Hospital Affiliated to Shanghai University of Traditional Chinese Medicine, CHINA

## Abstract

**Objective:**

To report the clinical outcomes of canine ilial body fractures stabilized with a newly designed, anatomically 3.5 mm Steelvet Iliac Locking Plate.

**Study design:**

Retrospective case series.

**Animals:**

Ten client-owned dogs treated between 2023 and 2024.

**Methods:**

Medical records and orthogonal pelvic radiographs were reviewed for signalment, fracture configuration, surgical findings, time to radiographic union, postoperative sacral index (SI), complications, and limb function. Continuous variables were summarized as median (interquartile range, IQR), mean ± standard deviation (SD), and range; categorical variables as counts and proportions with Wilson 95% confidence intervals (CIs).

**Results:**

Median age was 3.5 years (IQR 2.25–5.75; range 0.75–10) and median body weight 32.0 kg (IQR 28.3–38.5; range 21–52). Median time to radiographic union was 60 days (IQR 60–62.5; range 60–97). Six of ten dogs (60%; 95% CI 31.3–83.2%) achieved union by 60 days. One dog (10%; 95% CI 1.8–40.4%) developed a cutaneous fistula that resolved within seven days of plate removal after fracture healing. The SI was within normal limits in all dogs at final follow-up, and limb function was normal in 10/10 dogs.

**Conclusions:**

In this small case series, stabilization of canine ilial body fractures with the dedicated 3.5 mm Steelvet Iliac Locking Plate was associated with reliable bone healing, a low complication rate, and preserved pelvic canal dimensions. Clinical significance: A dedicated, anatomically contoured locking plate is a feasible option for fixation of canine ilial body fractures, conforming to the iliac surface with no or minimal intraoperative contouring and allowing straightforward application. Larger comparative studies are warranted.

## Introduction

Pelvic fractures are a prevalent concern in companion animals, representing approximately 25% of all fractures in cats and 16% in dogs [1]. The majority of these injuries are attributed to motor vehicle accidents; however, other significant causes include high-energy incidents such as falls from heights and blunt trauma [1–3].

The outcomes of surgical management for ilial body fractures are reported to be excellent, typically leading to a quick return to function [4]. Complications arising from the repair of ilial fractures may be linked to insufficient fixation strength. This inadequacy can lead to several issues, including nonunion of the fracture, the risk of implant infection, and potential fracture of the bone plate [3–5].

A range of plate types is employed for the stabilization of ilial fractures, including dynamic compression plates (DCP), locking plates, cuttable plates, T plates, mini plates, reconstruction plates, tibial plateau leveling osteotomy plates, and double plates [2–4,6–13]. Published papers demonstrate that conventional plates have a twentyfold higher likelihood of implant failure compared to locking plates [4]. Locking and non-locking constructs differ in the way the screws engage the implant. In a locking plate, the screw heads thread directly into the plate holes, creating a fixed-angle construct in which the screw–plate interface is stable independent of friction between the plate and the underlying bone. Conventional non-locking plates, by contrast, rely more heavily on compression of the plate against the bone surface, generated as the screws are tightened, to achieve stability. Because the fixed-angle interface resists screw loosening and toggling, locking constructs are associated with a lower risk of implant failure, which is a principal advantage of the plate evaluated here.

Importantly, none of these plates were specifically designed to treat ilial fractures. To the authors’ knowledge, the only study referenced in the existing literature that pertains to a dedicated plate for the treatment of ilial fractures is that conducted by Moi et al. [14]. This preliminary investigation used finite element methods to explore the design of a plate intended specifically for ilial fractures. However, it is important to note that this plate was never manufactured and has not been evaluated in cadaveric studies or living patients. The aim of the present study was to assess the clinical outcomes of canine ilial body fractures treated with a new dedicated 3.5 mm Steelvet Iliac Locking Plate. The plate was manufactured from titanium alloy (Ti-6Al-4V ELI); compared with stainless steel, titanium and its alloys are favored for orthopedic implants because of their high strength, corrosion resistance, and biocompatibility [15]. We hypothesized that the use of this dedicated plate would be associated with a low complication rate comparable to or lower than that reported in previous studies.

## Materials and methods

Every client upon admission to the Veterinary Medical Center of the University of Tennessee; by signing a form, the client authorizes the University of Tennessee College of Veterinary Medicine to use their pet's information for research purposes. Informed owner consent was therefore obtained for all cases.

### Implant design and development

Seven computed tomography (CT) scans of dogs weighing 25–30 kg were used to develop a statistical shape model (SSM) of the ilium, in order to derive an anatomically representative contour for a dedicated ilial plate.

Following segmentation of the iliac bone from each CT scan, an STL file of the right ilium was generated and imported into ShapeWorks software for shape analysis.

To construct the SSM, the iliac surfaces of the seven ilia were superimposed and homologous landmarks identified on the bone surfaces were used for SSM calculations. Morphological variation in shape and size among specimens was characterized and used to inform the design of the dedicated plate [16–18]. The statistical shape model was used to derive an anatomically representative contour so that the resulting plate would conform closely to the native iliac surface, requiring no or minimal intraoperative contouring for dogs over 25 kg, since the anatomy of the ilium will be similar in most dogs.

The plate project was developed using SolidWorks™ 3D CAD design software to create a universal shape compatible with the final model of the ilium derived from the SSM process. This plate extends from the iliac wing to the cranial region of the acetabulum. It has been specifically designed to provide extensive contact with the iliac body, thereby enabling the utilization of multiple screws within the iliac wing (Fig 1).

**Fig 1:**
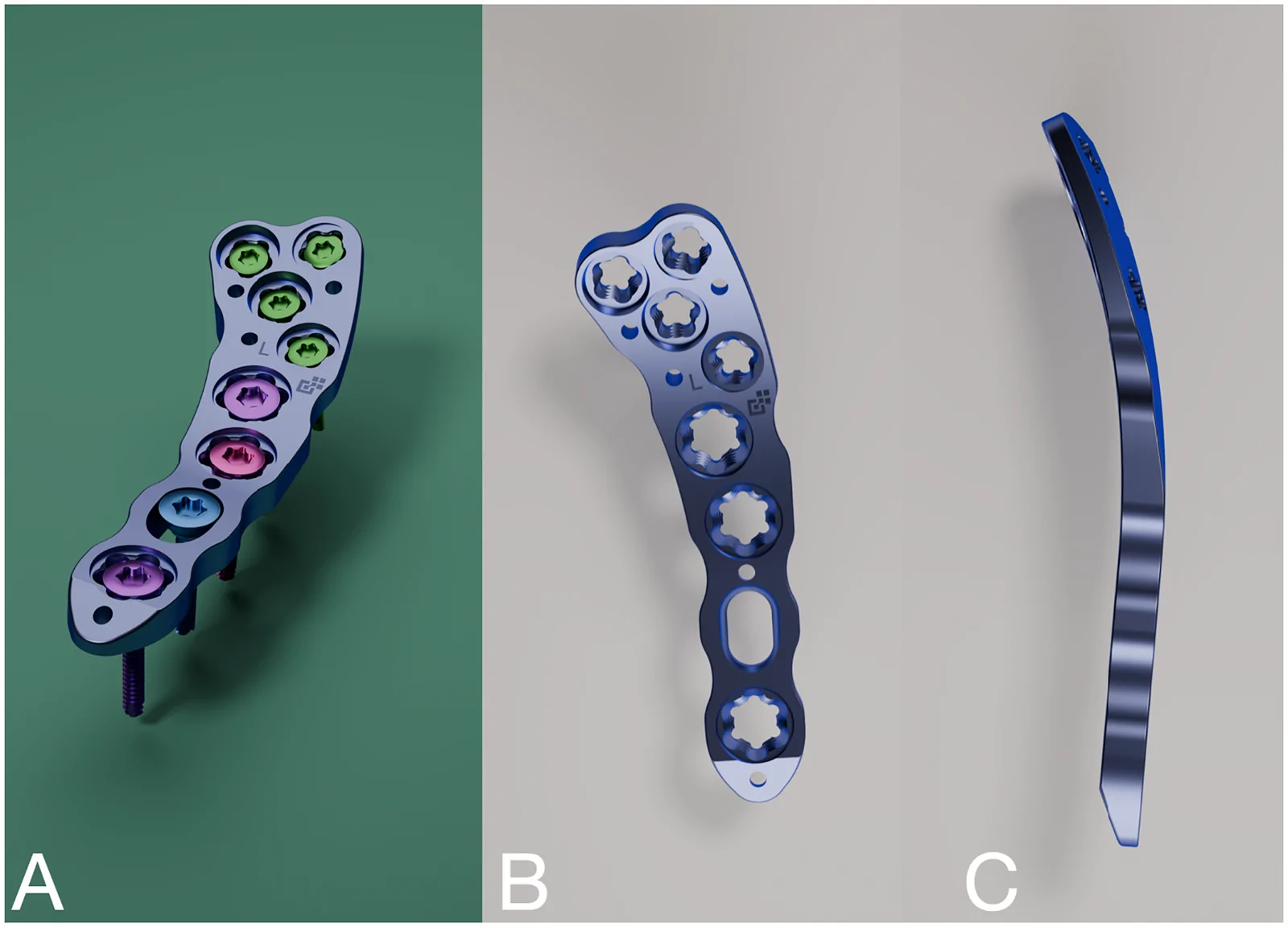
Three-dimensional views of the ilial Steelvet anatomically plate. Cranial **(A, B)** and lateral **(C)** views illustrate the anatomical contouring and screw hole configuration. Screw colors denote the hybrid construct: green, 2.5 mm locking; purple, 3.5 mm locking; red, 4.2 mm locking; and blue, 3.5 mm cortical.

The ilial plate is a hybrid implant. The proximal portion (plate head) accepts 2.5 mm or 3.5 mm locking screws, and the plate shaft accepts 3.5 mm or 4.2 mm locking screws. Variable-angle locking holes allow angulation of the locking screws up to 15° from the neutral axis in any direction, enhancing surgical versatility and stability (Fig 2). The plate also incorporates a non-locking compression hole that allows fracture or osteotomy compression during plate application (Fig 3).

**Fig 2:**
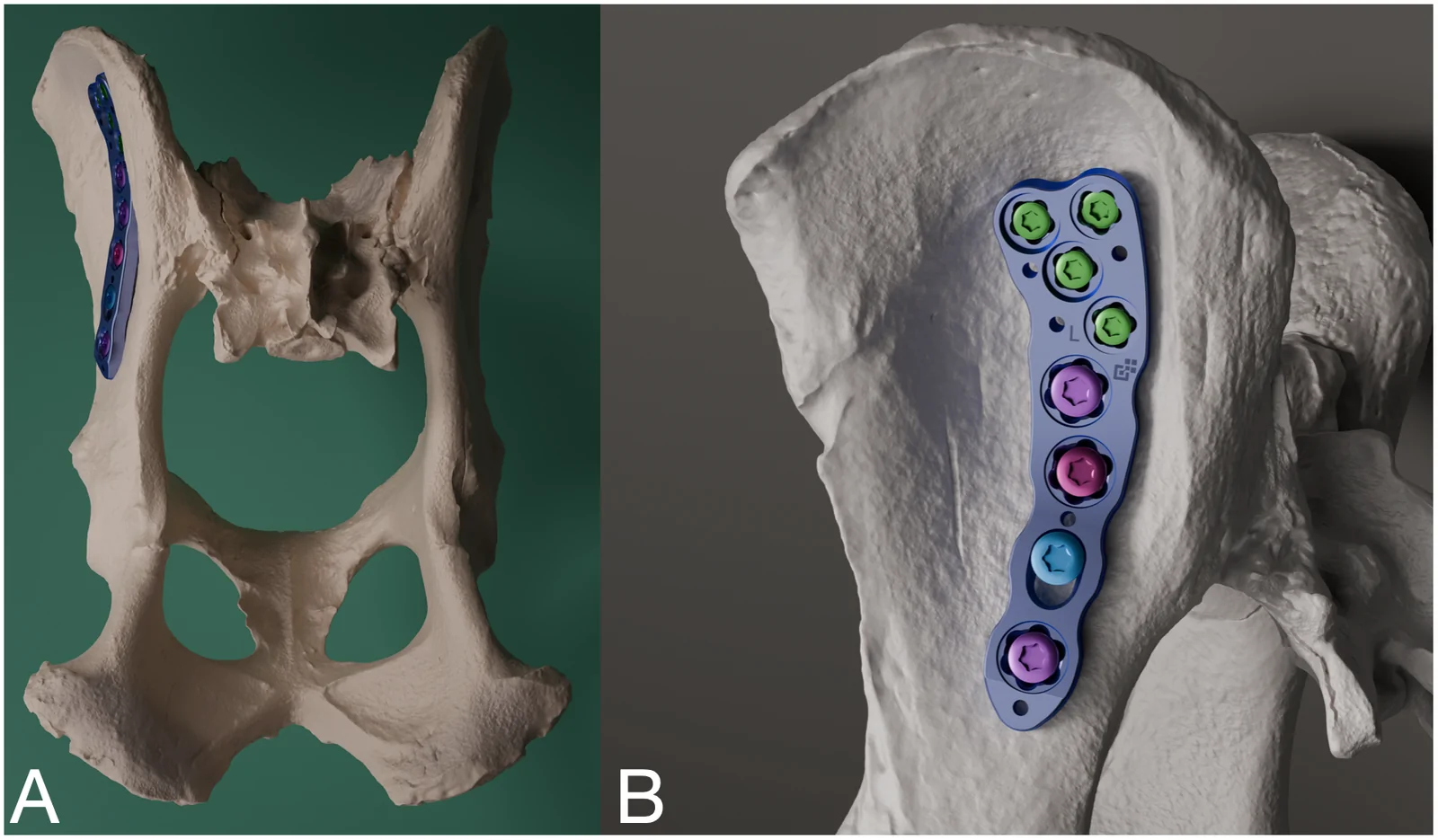
Three-dimensional rendering of the ilial Steelvet anatomically plate and its anatomical positioning on a canine pelvis model. The plate is shown along the lateral ilial surface in dorsal **(A)** and lateral **(B)** views, illustrating the relationship between the dorsal flange and the ilial body.

**Fig 3:**
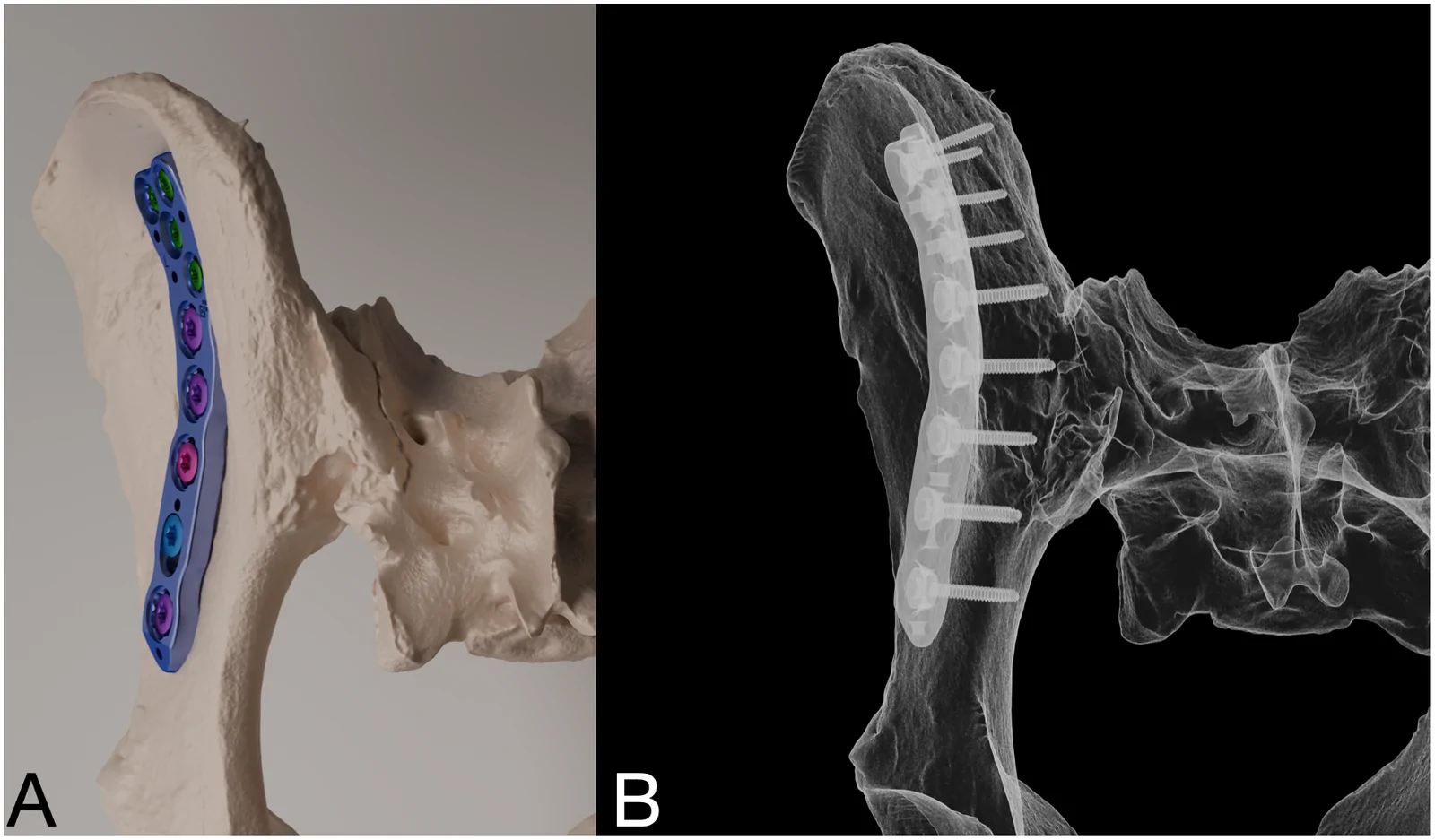
Application of the ilial Steelvet anatomically plate on a canine pelvis model. **(A)** Dorsal view of the pelvis with the plate seated along the dorsolateral aspect of the left ilial wing, extending from the iliac crest cranially to the body of the ilium caudally. **(B)** A x-ray of same canine pelvis model showing the hybrid screw construct in cranial-to-caudal sequence: four 2.5 mm locking screws in the cranial cluster engaging the thin cortical bone of the iliac wing in a dorsoventral trajectory; two 3.5 mm locking screws and one 4.2 mm locking screw directed mediolaterally across the ilial body toward the sacral articulation; a single 3.5 mm cortical screw placed as a lag/positioning screw at the plate waist; and a caudal 3.5 mm locking screw anchoring the plate near the body of the ilium. Screw trajectories diverge to maximize cortical purchase and avoid the sacroiliac joint and acetabulum.

### Technical description of the 3.5 mm steelvet iliac locking plate

The 3.5 mm Steelvet Iliac Locking Plate is a hybrid, dedicated implant developed with attention to clinical and anatomical requirements for the iliac wing region.

The plate is anatomically contoured, with side-specific (left and right) variants, and is available in models with 4 holes in the proximal region (plate head) that use Ø2.5mm or 3.5 mm locking screws and 4 and 5 holes in the plate shaft that use Ø3.5mm or Ø4.2mm locking screws. The proximal width is 20 mm and the distal width 10 mm, with a thickness of 3.5 mm; the plate is available in 60 mm and 70 mm lengths. The plate is manufactured from titanium alloy Ti-6Al-4V ELI in accordance with ASTM F136. The plate is produced by subtractive manufacturing using a laser cutter and a milling machine, as is conventional for commercially available orthopedic plates. The 3.5 mm Steelvet Iliac Locking Plate is commercially available (Steelvet).

The 3.5 mm Steelvet Iliac Locking Plate also has small accessory holes along its body that serve as provisional fixation points to the bone prior to screw insertion. Used with stopped guide wires (Guide Wires with Stops), these holes facilitate fracture reduction and stabilize the plate in the desired anatomical position without the need to hold it manually during screw placement.

All locking and non-locking screws have a self-cutting tip and a hexalobular drive interface. The hexalobular interface reduces wear at the screwdriver tip, improves screw retention on the driver, and allows a low-profile screw head that sits flush with the plate surface after placement.

### Case selection

Medical records were reviewed for all client-owned dogs that underwent surgical repair of an ilial body fracture using the dedicated 3.5 mm Steelvet Iliac Locking Plate between 2023 and 2024 (n = 10). Dogs were included if they had an ilial body fracture treated with the anatomically 3.5 mm Steelvet Iliac Locking Plate during the study period and had postoperative clinical and radiographic follow-up records available; cases would have been excluded only where such follow-up records were unavailable or the dog was too small or too large for the implant sizes available for the study. All dogs treated with the implant during this period met these criteria and were included, and no case was selected or omitted on the basis of clinical outcome. Cases were followed clinically and radiographically until radiographic union was documented or a complication occurred.

### Surgical technique

All procedures consisted of open reduction and internal fixation through a standardized lateral approach, using a gluteal roll-up to expose the ilial body as previously described [16]. The dedicated 3.5 mm Steelvet Iliac Locking Plate was used for fracture fixation in all cases.

### Outcome measures

Pelvic alignment, fracture apposition, implant integrity and position, and healing at the fracture gap were assessed on orthogonal immediate postoperative and follow-up radiographs by a single investigator. Each parameter was categorized as anatomic, near anatomic, good, fair, or poor according to the criteria reported by Petrovsky et al. [4].

The sacral index (SI) was established as an objective metric for assessing alterations in pelvic alignment by calculating the ratio obtained from ventrodorsal radiographs (X-rays). The formula employed for this purpose is SI = AW / SW (Narrowest Pelvic Canal Width / Width of Cranial Sacrum) [19].

The sacral index was assessed using the immediate postoperative radiograph and the final follow-up radiograph. The index was considered abnormal if it was below 0.9, and indicative of significant pelvic canal narrowing attributable to malunion if it was below 0.76 [19].

## Results

Ten dogs that underwent surgical repair of an ilial body fracture were included (Table 1). Breeds represented were German Shepherd, Great Pyrenees, Boxer, Labrador Retriever, and mixed breed. Median age was 3.5 years (range 9 months to 10 years) and median body weight was 32 kg (range 21–52 kg). Given the open enrollment criteria, every dog treated with the anatomically 3.5 mm Steelvet Iliac Locking Plate during the study period was included; no dog was excluded or failed to meet the enrollment criteria.

**Table 1:** Signalment, fracture characteristics, and outcomes of the ten dogs with ilial body fractures treated with the dedicated 3.5 mm Steelvet Iliac Locking Plate.

Breed	Age (years)	Weight (kg)	Time to healing (days)	Complications	Treatment	Fracture type	Limb function
German Shepherd	2	32	97	No	Ilial plate	Short oblique	Normal
Mixed breed	8	28	61	No	Ilial plate	Short oblique	Normal
Great Pyrenees	1	43	63	Yes (infected); explanted at 3 months	Ilial plate	Comminuted	Normal
Mixed breed	4	26	60	No	Ilial plate	Short oblique	Normal
Boxer	3	39	60	No	Ilial plate	Short oblique	Normal
Mixed breed	0.75	21	70	No	Ilial plate	Short oblique	Normal
Labrador Retriever	3	32	60	No	Ilial plate	Comminuted	Normal
Mixed breed	10	52	60	No	Ilial plate + FHO	Comminuted	Normal
Labrador Retriever	5	37	60	No	Ilial plate	Short oblique	Normal
Mixed breed	6	29	60	No	Ilial plate	Short oblique	Normal

Fracture healing time, defined as the number of days from surgery to radiographic union, showed some variability among patients. Six of ten dogs (60%) achieved radiographic union by 60 days, and all dogs achieved union by 97 days. Two cases showed extended healing durations: one German Shepherd healed in 97 days and one mixed-breed dog in 70 days.

No major intraoperative or early postoperative complications were observed. One dog (10%; 95% CI 1.8–40.4%) developed a cutaneous fistula adjacent to the surgical incision. There was no radiographic or clinical evidence of osteomyelitis; the plate was explanted after the fracture had healed, and the fistula resolved within seven days of plate removal. The sacral index was within normal limits (≥ 0.9) in all dogs at final follow-up.

Follow-up for each dog extended until the final radiographs documenting fracture healing, or until a complication arose, as occurred in one dog. All dogs showed normal weight-bearing at the 60-day recheck. At the most recent follow-up, no animal exhibited signs of long-term constipation, abnormal micturition, or pain on hip range-of-motion examination, and all dogs maintained normal ambulation.

### Statistical analysis

Given the retrospective design and small sample size, analyses were descriptive. Continuous variables (age, body weight, time to radiographic union) were summarized as median (interquartile range, IQR), mean ± standard deviation (SD), and range. Specifically, age was summarized as median 3.5 years (IQR 2.25–5.75), mean 4.28 ± 3.01, range 0.75–10 years; body weight as median 32.0 kg (IQR 28.3–38.5), mean 33.9 ± 9.1, range 21–52 kg; and time to radiographic union as median 60 days (IQR 60–62.5), mean 65.1 ± 11.6, range 60–97 days

Categorical variables (fracture configuration, complication occurrence, limb function) were summarized as counts and proportions. The proportion of dogs that healed by 60 days was calculated as 6/10 (60%), with a Wilson 95% confidence interval (CI) of 31.3–83.2%; the proportion with postoperative complications was 1/10 (10.0%), with a Wilson 95% CI of 1.8–40.4%. Limb function at latest followup was recorded as normal in 10/10 dogs.

Healing times were also summarized by fracture configuration (short oblique vs comminuted) using descriptive statistics only: comminuted (n = 3): mean 61.0 days (range 60–63), median 60; short oblique (n = 7): mean 66.9 days (range 60–97), median 60. No formal statistical comparisons were performed, and no hypothesis testing was undertaken, as the study was not powered for inferential analysis. All analyses were conducted at the case level.

## Discussion

The present study evaluated the fracture healing outcomes in 10 canine patients treated surgically for ilial fractures (Fig 4) using internal fixation (Fig 5). The patient population was diverse in terms of age, breed, and body weight, as was seen in other studies [4], reflecting the broad applicability of the surgical technique across a diversity of patients.

**Fig 4:**
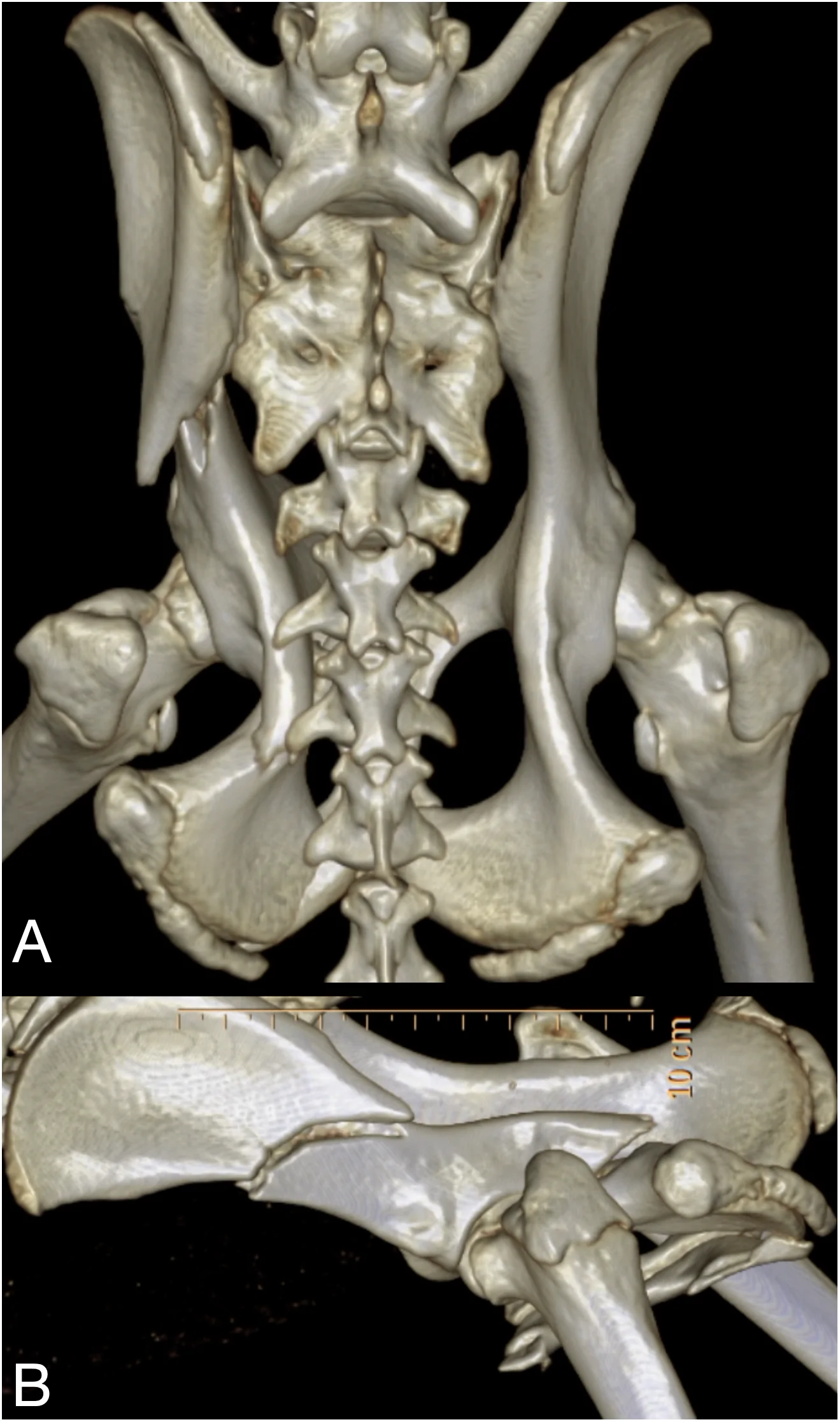
Computed tomography of a dog with short oblique ilial and ischial fractures. Multiplanar reconstruction shown in the dorsal plane **(A)** and sagittal plane **(B)**.

**Fig 5:**
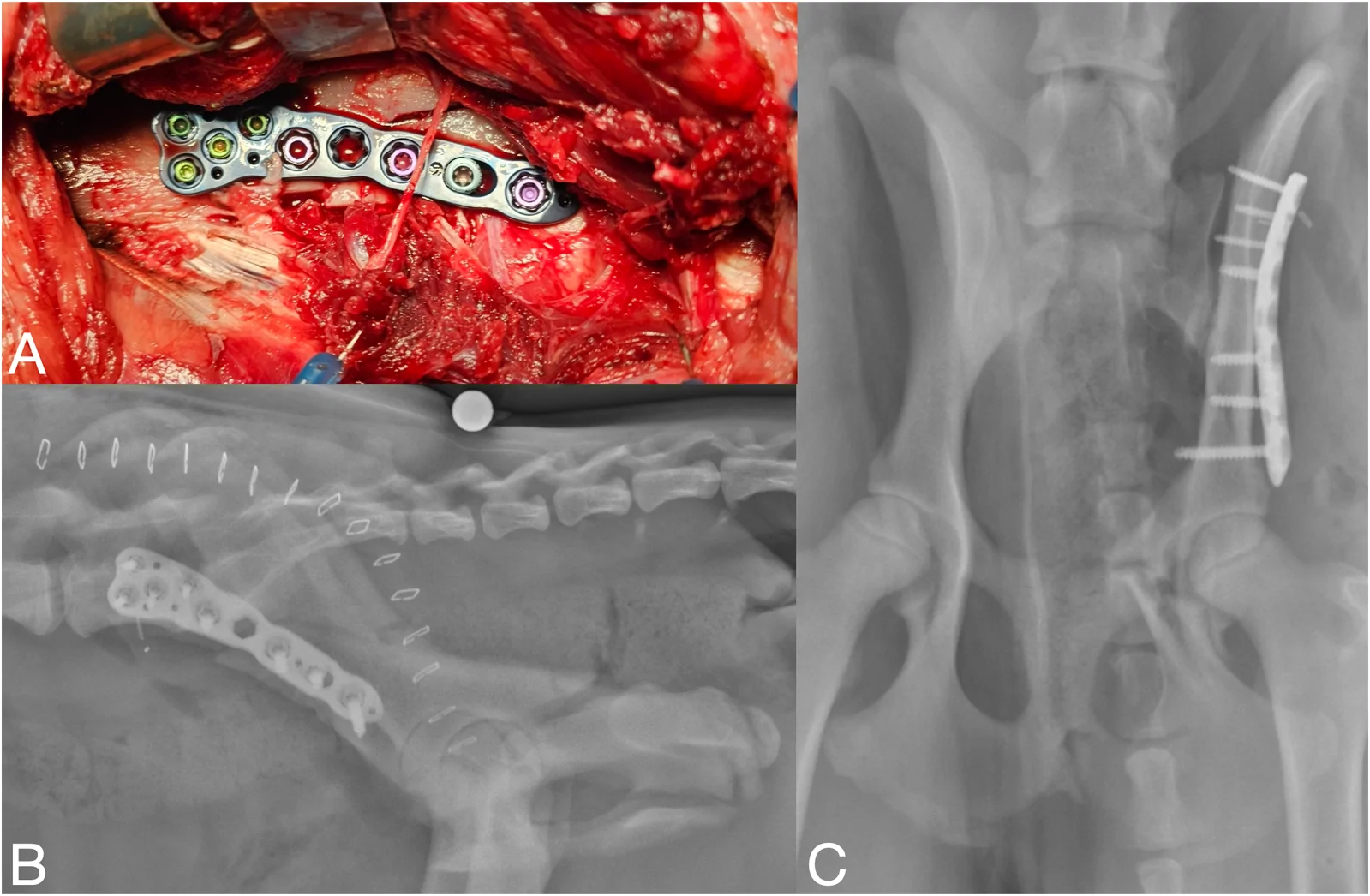
Intraoperative and immediate post-surgery imaging of a dog with a short oblique ilial fracture. **(A)** Intraoperative image showing fracture fixation with a anatomically ilial plate and seven screws (four green 2.5 mm, three purple 3.5 mm, and one blue cortical 3.5 mm). Laterolateral **(B)** and ventrodorsal **(C)** immediate post-surgery radiographs.

The healing time across most patients was favorable, with 6 of 10 dogs achieving radiographic union by 60 days postoperatively and all dogs by 97 days (Fig 6). These findings suggest that the fixation method employed provided adequate biomechanical stability to allow early and consistent healing, regardless of breed or weight, within a typical recovery window. The high rate of consolidation was expected in pelvic fractures [20], particularly following surgical management. The pelvis is encircled by muscles that effectively stabilize the region and supply blood, which contributes to the high rate of bone union, whether surgical or conservative management is employed [21,22].

**Fig 6:**
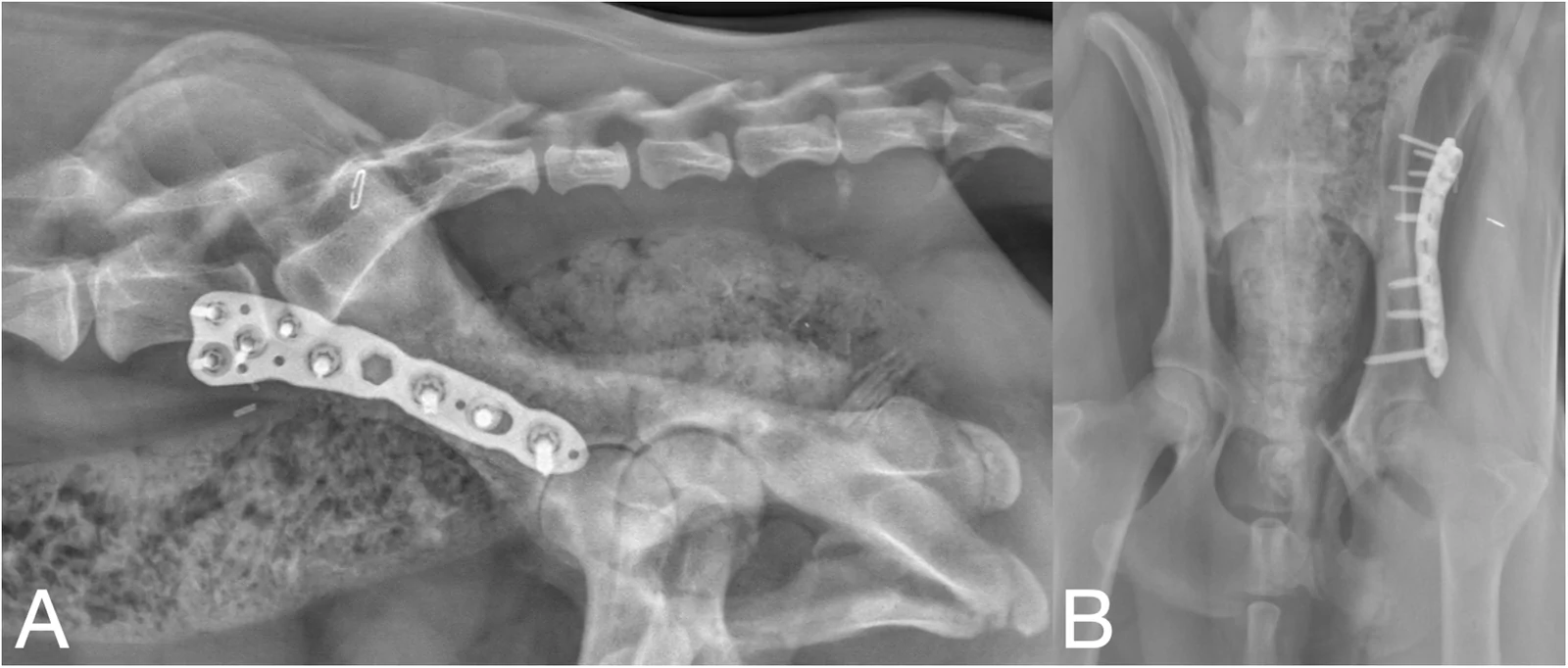
Sixty-day post-surgery follow-up radiographs of a dog with short oblique ilial fracture. Laterolateral **(A)** and ventrodorsal **(B)** radiographs obtained 60 days post-surgery. Ilial healing is evident on both views.

Although issues such as infection, nonunion, and plate failure have been reported following repair of ilial fractures [3–5], no implant-related mechanical complications and no nonunions were observed in the present series. One dog developed a cutaneous fistula that required plate removal; the fracture had already healed by the time of explantation, and the fistula resolved within seven days. These findings support the safety profile of the implant, although the limited sample size precludes firm conclusions.

One of the major concerns in ilial fracture repair is implant failure, often resulting from inadequate fixation strength [4] or biological complications such as infection. In this study, none of the cases experienced implant-related complications, even though two cases showed minor delayed union (70 and 97 days).

When contextualized against traditional fixation strategies, the outcomes of the present series compare favorably with those previously reported for conventional constructs. With respect to healing time, radiographic union in our cases (median 60 days; all dogs by 97 days) was consistent with the intervals reported for surgically managed ilial fractures repaired with dynamic compression, reconstruction, and standard locking plates [4,20]. With respect to implant failure and screw loosening, conventional non-locking plates carry an approximately twentyfold higher likelihood of failure than locking constructs [4], and implant loosening or failure remains the most common complication of ilial fracture repair, reported in up to 62% of cases [19]; by contrast, no implant-related mechanical failure, screw loosening, or nonunion occurred in the present series. With respect to pelvic canal narrowing, the sacral index remained within normal limits in all dogs, whereas malunion and canal stenosis may follow less anatomically precise reduction [19]. With respect to infection, a single cutaneous fistula (10%) resolved after plate removal, without radiographic or clinical evidence of osteomyelitis, a rate comparable to the surgical-site complications reported for other plating techniques [4]. Finally, with respect to return to function, all dogs were weight-bearing within the first postoperative week and regained normal limb function, mirroring the rapid recovery described after surgical management of pelvic fractures [20] and contrasting with the sub-optimal long-term function associated with conservative management [20]. Nevertheless, because the present study lacked a concurrent comparator group, these comparisons remain observational, and the anatomically contoured design cannot be concluded to be superior to established fixation methods on the basis of the current data.

Fractures of the ilial body are a significant concern in small animal orthopedic surgery [1] due to their potential to compromise pelvic stability and neurovascular structures [4]. The results of this study reinforce the role of surgical fixation in restoring function and minimizing complications. In our opinion, the anatomically dedicated shape of the plate used in this study facilitated accurate fracture reduction and may reduce the likelihood of complications such as pelvic canal narrowing.

Pelvic canal narrowing and malalignment are potential long-term sequelae of ilial fractures, which can lead to issues such as constipation or nerve entrapment. This highlights the importance of precise fracture reduction and stable fixation techniques to prevent secondary complications. Custom-made implants developed based on specific anatomical requirements have decreased complication rates in veterinary surgery, like those used in TPLO, TTA, and CBLO, and it has stimulated some studies to achieve custom-made implants for pelvic fracture [14], like those utilized in this study.

Fracture configuration also played a role in healing outcomes. Short oblique fractures tended to heal more predictably than comminuted fractures, likely due to the greater difficulty in achieving stable fixation in the latter. The biomechanical properties of the ilium, including cortical thickness and load-bearing capacity, should be considered when selecting plating techniques. In some cases, additional fixation strategies such as secondary plates or sacral screws may improve overall construct stability.

Titanium alloy (Ti-6Al-4V ELI) was selected for this implant primarily for its biocompatibility with bone and its manufacturability. Compared with stainless steel, titanium and its alloys are generally favored for orthopedic implants because of their high strength, corrosion resistance, and biocompatibility [15]. Titanium also has a lower elastic modulus—approximately half that of 316L stainless steel—which more closely approximates that of bone and may reduce stress shielding [15,23]. In addition, titanium is well suited to precise subtractive fabrication by laser cutting and milling, which facilitated production of the anatomically contoured plate used in this study. These material properties are consistent with the low complication rate observed in the present series, although no adverse material-related reaction was specifically evaluated.

The proposed advantage of this anatomically dedicated implant merits comment. Because the present study did not include a concurrent comparator group, we cannot formally attest to a specific biological or mechanical benefit over conventional plates. Nevertheless, several practical observations were consistent across the series. All plates conformed to the iliac surface and required no intraoperative contouring, and the implant was easy to apply. The anatomically dedicated shape also appeared, anecdotally, to allow a more favorable screw trajectory and a higher screw density in each bone fragment than would typically be achievable with a straight conventional plate, which may enhance fixation in the relatively thin iliac wing. These impressions are qualitative and require confirmation in controlled comparative studies. Notably, the limitations of straight conventional plates for ilial fixation have prompted others to repurpose non-dedicated implants to increase screw purchase; for example, tibial plateau leveling osteotomy (TPLO) plates have been used in place of a straight plate to accommodate additional screws in the iliac wing [12].

The study was limited by its small sample size, its retrospective design, and the absence of validated objective functional outcome measures; functional assessment was based on normal weight-bearing and clinical orthopedic examination, both of which are relatively subjective. Healing time was assessed radiographically, and long-term data on implant integrity and functional outcomes were not available. Future prospective research with a larger cohort should incorporate standardized objective outcome measures—such as kinetic gait analysis, force-plate evaluation, and validated orthopedic scoring systems—alongside standardized postoperative radiographic assessment to confirm and quantify these preliminary findings. Broader validation across a larger and more heterogeneous population remains desirable; in particular, giant and toy breeds at the extremes of the size range were not represented in the present series.

## Conclusion

This case series of 10 dogs with ilial body fractures, internal fixation with the anatomically dedicated 3.5 mm Steelvet Iliac Locking Plate was feasible and was associated with reliable bone healing, a low complication rate, and preserved pelvic canal dimensions. These findings should be interpreted as preliminary evidence of feasibility and safety rather than of superiority over established fixation methods; because the study lacked a concurrent comparator group, no conclusion can be drawn regarding the relative performance of this implant. Larger comparative and biomechanical studies, together with standardized postoperative monitoring, are required to confirm these observations and to define any advantage of the dedicated plate.
